# Exploring social perceptions of everyday smartglass use in Australia

**DOI:** 10.1371/journal.pone.0313001

**Published:** 2024-11-01

**Authors:** Fareed Kaviani, Ben Lyall, Sjaan Koppel

**Affiliations:** 1 The Emerging Technologies Research Lab, Monash University, Melbourne, Australia; 2 Monash University Accident Research Centre, Monash University, Melbourne, Australia; University of Konstanz: Universitat Konstanz, GERMANY

## Abstract

Smartglasses like Ray-Ban Stories by Meta are now commercially available, offering users features like photography, videography, music playback, phone calls, and content sharing. While existing research identifies barriers to adoption, no study has investigated the social acceptability of these commercially available devices. This is crucial because devices like Ray-Ban Stories are considered precursors to Augmented Reality-enabled smartglasses, and understanding current public perceptions is vital before further advancements. This study aimed to examine the social acceptability of everyday smartglass use. An online survey recruited 1037 Australian residents aged 18+ (58.6% owners, n = 608; 41.4% non-owners, n = 429). The WEAR scale assessed social acceptability. Owners perceived the device as aligning with their desired self-image, while non-owners expressed stronger concerns regarding privacy, anti-social behaviour, and potential harm. The WEAR scores highlight contrasting expectations between owners and non-owners regarding appropriate technology use, suggesting a potential source of social tension. Further research is needed to understand how individuals negotiate the use of these devices in public spaces.

## 1. Consumer smartglasses

The release of Meta’s Ray-Ban Stories in September 2021 in Australia demonstrates the rapid advancement in smartglass design and functionality since Google Glass, the first personal-use smartglass product announced for commercial use in 2013 [1]. While Meta’s Ray-Ban Stories are not the first smartglass technology to enter the commercial market, no other smartglass device has achieved such widespread coverage across major eyewear retail stores in Australia, catching the attention of Australia’s privacy watchdog, the Office of the Australian Information Commissioner (OAIC) [2].

Smartglasses are a type of wearable technology designed for "continuous use and multitasking" and have the potential to revolutionise how we interact with the world around us [3]. Wearables are computing devices that can be worn on the body, including those requiring additional computing functionality [4]. They often operate autonomously, without an internet connection, and take the form of non-smart accessories like bracelets, shoes, or glasses [5, 6]. The visual and recording capabilities of smartglasses further categorise them alongside other emerging camera-based technologies like drones and driverless cars, which can record and analyse images on behalf of users [7]. Ray-Ban Stories do not have display functionality, however, they are seen as a stepping stone towards fully realised augmented-reality glasses. The increased mediation of the physical through digital technologies raises critical questions about self-perception, privacy, and ethical behaviour, highlighting the urgent need for research into how these devices are currently being used and how non-users perceive their social acceptability.

### 1.1. Researching public use of commercial smartglasses

When connected to a smartphone, Meta’s Ray-Ban Stories allows users to take pictures, record videos, play music, make and receive phone calls, and share content across Meta’s platforms. The ethical and privacy implications of the widespread use of such devices, particularly the potential for covert recording and sharing of private conversations and activities, give rise to serious concerns about data privacy, heightened surveillance, and monitoring, ultimately impacting public safety and well-being [8]. The limited large-scale studies on user interactions with these devices and public acceptance of wearing them is primarily due to their lack of commercial success to date [9]. This lack of research also hinders efforts to establish a standardised definition of smartglasses [10].

Research on the everyday use of smartglasses is limited, primarily taking place in specialised settings such as in hospitals, where the technology is transforming the healthcare sector through advancing surgical and clinical practices [5, 10]. Outside these settings, smartglass research is predominantly consumer focused, examining factors that may influence acceptance and increase adoption. For instance, emphasising the role of individual personality traits in the adoption of wearable technology, Rauschnabel et al. [11] found openness increased consumer awareness of the device, while the potential of smartglasses for functional benefits and social conformity significantly influenced adoption intentions. Basoglu et al.’s [12] study notably emphasised the role of social influence, extending the Technology Acceptance Model (TAM) to include factors such as peer influence alongside perceived usefulness and ease of use. While Rauschnabel et al. [11] underscored the moderating effects of personality traits, Basoglu et al. [12] provided a broader model incorporating social and individual factors to explain adoption behaviour. However, their extended TAM does not account for the importance of aesthetic qualities as a determining factor.

Indeed, Meta’s Ray-Ban Stories are almost indistinguishable from traditional Ray-Ban sunglasses, differing significantly in appearance and functionality from those used in specialised settings or from older devices like Google Glass. It could be argued that the integration of fashion and technology facilitated the success of this new generation of smartglasses. The rapid advancements in smartglass technology highlight the critical need to understand how these devices are perceived and used in everyday contexts. Unfortunately, research outside of the applied sciences primarily focuses on marketing and consumer adoption, inadvertently normalising smartglass technologies. This is exemplified by Meta’s response to the Australian Competition and Consumer Commission (ACCC) inquiry, where they used AR/VR investment trends as justification for their practices [13]. However, recent reviews of Australia’s Privacy Act (1988) reflect an increased concern for the diverse forms of data collected, traded, and analysed from wearable sensors, including health, biometric, and geolocation information [14]. This demonstrates a growing awareness of the potential impact of these technologies and the need for regulations to protect privacy and security.

Despite the potential impact on urban governance, user safety, and privacy, large scale research exploring how the unique aspects of this new generation of smartglasses impacts both users and non users is scarce. This includes how smartglasses influence our sense of self, social interactions, movement in public spaces, and everyday life. For example, research suggests that contextual factors, such as using smartglasses during activities susceptible to misuse, can affect their social acceptability [15]. Additionally, a person’s social circle and personal privacy concerns can influence perceptions, as wearers of mobile devices are often anticipated to belong to specific, stereotypical user groups [16, 17]. Bolesnikov [18] identified a lack of research on the perceived acceptability of wearables on non-privileged bodies, highlighting how the social acceptability of the technology can be diverse and polarised, reflecting user characteristics and specific usage contexts. This demonstrates how new technologies disproportionately impact individuals, potentially (re)producing social and structural inequalities. Hofmann [3] argues that the heterogeneity of issues surrounding digital technology mirrors the diversity of potential smartglass users. Therefore, device assessments should occur within specific contexts of use to develop tailored recommendations relevant to contemporary users and challenges. Research must not only consider technological advancements and consumer perspectives on smartglasses but also the social implications for individuals, communities, and society as a whole. While understanding ownership prevalence and user types and behaviours is crucial, this study aims to serve as a springboard for future investigations into how Australian users perceive the social acceptability of smartglasses.

### 1.2. WEAR scale

Kelly and Gilbert [19] developed the WEAR (Wearable Acceptability Range) scale to assess the social acceptability of various wearable devices or prototypes. Previous research has employed this scale to evaluate the social acceptability of six different wearables, including a wrist-worn smartphone, wireless earbuds, and a brain-sensing headband [19]. The WEAR scale was designed to enable the wearables industry to more accurately predict the human factors influencing wearables’ social acceptability during development and before product launch [19]. It analyses how well the technology fulfils aspirational desires, such as belonging to a social group or enhancing self-image, and assesses the level of social anxieties associated with wearable technology, such as privacy concerns, appropriateness of use, and potential safety risks.

### 1.3. This study

Research has demonstrated that technology ownership or previous experience with a technology significantly influences attitudes towards that technology. Ownership may foster a sense of familiarity and attachment, a phenomenon called the endowment effect, which suggests that people ascribe more value to things merely because they own them [20]. Similarly, individuals who own a particular technology may overlook or justify the negative behaviours associated with that technology. For example, smartphone owners with a greater attachment to their devices expressed greater enthusiasm for the convenience it provided, however, they were also more likely to engage in problematic behaviours such as dangerous or prohibited smartphone use. Furthermore, smartphone owners who used their devices illegally while driving engaged in risk-compensatory strategies or rationalised their transgressions to mitigate the severity of their behaviours [21].

Yet, no research exists that uses the WEAR scale to examine the perceptions and sentiments of both smartglass owners and non-owners to determine if ownership is a significant factor in an individual’s acceptance of these technologies. Similarly, while it is well known that younger people are more likely than older individuals to be avid adopters of technology [10], the role of gender in determining the adoption and use of new and emerging technologies is less concrete. Despite this, research showed that both age and gender impact how individuals engage with mobile technologies. For instance, research into problematic mobile phone use showed that younger people and men were more likely than older individuals and women to use their smartphones in a manner considered dangerous or when it was prohibited to do so [22]. Therefore, this study applies the WEAR scale to smartglass owners and non-owners to compare their perceptions, sentiments, and readiness towards the technology. It examines how age, ownership, and gender influence these perceptions and usage patterns, focusing on the potential benefits and risks related to self-image, social interaction, and privacy concerns, while considering individual differences in technology adoption.

## 2. Method

This section describes the methods used in the study, including participant selection, data collection, and measurement instruments.

### 2.1. Participants

Participants had to be 18 years of age or older and reside in Australia to participate in the survey. The survey took approximately 10–15 minutes to complete.

### 2.2. Socio-demographic characteristics

Eligible participants provided information on their gender (*Male*, *Female*, *Non-binary*, *Prefer not to disclose*), age (in years), residential postcode, the highest level of completed education (*Primary*, *Intermediate*, *VCE / HSC*, *Technical / TAFE*, *Diploma*, *Undergraduate*, *Postgraduate*, *Other*), current yearly household income (*less than $25*,*000*, *25*,*001 - $50*,*000*, *$50*,*001 - $75*,*000*, *$75*,*001 - $100*,*000*, *$100*,*001 - $125*,*000*, *$125*,*001 - $150*,*000*, *$150*,*001 - $175*,*000*, *$175*,*001 - $200*,*000*, *$200*,*001 - $250*,*000*, *more than $250*,*001*, *Prefer not to disclose*), ethnicity grouping (*Aboriginal or Torres Strait Islander*, *Asian*, *Black or African American*, *Middle Eastern*, *Native Hawaiian or Other Pacific Islander*, *Caucasian*, *Other*, *Prefer not to disclose*) and whether they are neurodiverse (*Yes*, *No*, *Prefer not to disclose*).

### 2.3. Materials

The online survey recruitment period commenced on 30/08/2022 and ended on October 7, 2022. Qualtrics was used to develop and administer the survey. Before commencing the survey, participants reviewed an explanatory statement and consent form (S1 File) that contained the survey eligibility criteria (age 18 or older, residing in Australia). Participants provided their informed consent to participate by selecting “Yes, I consent.” Participants that selected “no” were taken to the end of the survey. The survey included questions about familiarity with smartglasses, current smartglass ownership and use, perceptions and sentiments towards smartglasses (measured by the WEAR scale), and general willingness to try new technologies (measured by the Personal Innovativeness scale).

#### 2.3.1. Smartglass use and familiarity

**Fig 1:**
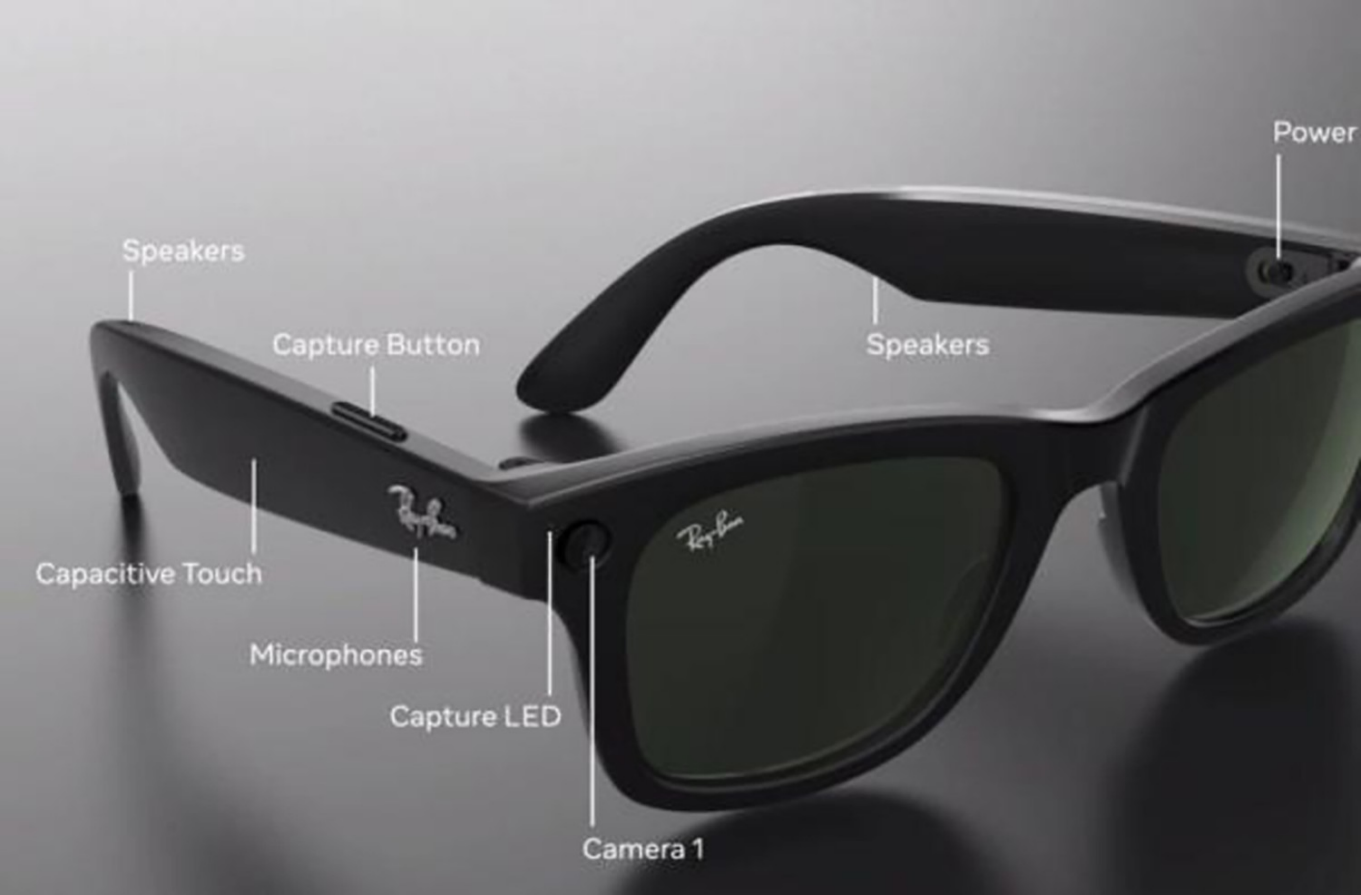
Ray-Ban stories. Source [8].

The online survey displayed an image of Ray-Ban Stories (Fig 1) and provided detailed information about its functionality: *“It is important to note that commercially available smartglasses do not currently include augmented reality displays (digital images displayed on the lens)*. *When connected to a smartphone*, the eyewear’s in-built camera* allows wearers to take photos or videos and upload them directly to social media. *The speakers and microphone enable calls and listening to music*. *All functions can be engaged hands-free or by using the eyewear’s touch-sensitive surface*. *Click here for more information [*https://www.ray-ban.com/australia/discover-ray-ban-stories/clp] *There is a colour indicator on the front of the glasses that lights up when camera recording is in use”.

Participants answered questions about personal smartglass ownership: (*Do you own a pair of Ray-Ban Stories or any other commercially available smartglasses*?) and knowledge of others owning smartglasses: (*Do you personally know anyone that owns a pair of commercially available smartglasses such as Ray-Ban Stories or Spectacles by Snap*?). For participants who owned smartglasses, the survey displayed follow-up questions: Average daily use (*How many hours per day do you typically use your smartglasses*?); function usage (*Which functions do you use most often*?: *Photo*, *Video*, *Music*, *Calls*, *Other*); frequency of use (*How often do you use each function*?: *1 = Never Use*, *2 = Almost Never*, *3 = Sometimes*, *4 = Almost Every Time*, *5 = Frequently Use*). These participants were asked if they have ever used their smartglasses in a manner that would be considered dangerous, such as while driving or cycling *(1 = No*, *2 = Maybe*, *3 = Yes*), and if they have ever used them in a prohibited manner, i.e., recording without consent, recording in a prohibited area *(1 = No*, *2 = Maybe*, *3 = Yes*).

#### 2.3.2. Measuring perceptions of smartglasses

To measure participant sentiment and perception towards smartglasses, we employed the WEAR scale [23]. This validated 14-item scale (S1 File) assesses the social acceptability of smartglasses through two factors:

1. *Fulfilment of Aspirational Desires*: This factor examines if smartglasses satisfy desires related to social belonging, self-image, and enhancing capabilities.
2. *Absence of Social Fears*: This factor assesses the presence of anxieties associated with smartglasses, such as privacy concerns, appropriateness of use, and potential safety risks.

The scale presents items in a randomised order and uses a 6-point Likert scale for responses (*1 = strongly disagree*, *6 = strongly agree*). Following the scale’s instructions, some items were reversed, as agreement signified a lack of social acceptability. The mean participant scores ranged from 1 (extremely low social acceptability) to 6 (extremely high acceptability). In this study, the WEAR scale demonstrated excellent internal consistency (Cronbach alpha = 0.85).

#### 2.3.3. Measuring technology acceptance

To measure participants’ general openness and willingness to embrace new technologies, we utilised the 4-item Personal Innovativeness (PI) scale [24] (S1 File). Research has shown a link between a person’s innovativeness and their acceptance of new technologies [25]. The PI scale uses a 7-point Likert scale (*1 = strongly disagree*, *7 = strongly agree*) for responses. Following Agarwal & Prasad’s instructions, the third item (*"In general*, *I am hesitant to try out new information technologies"*) was reversed. Total scores were calculated by summing the individual item scores, with a minimum of 7 and a maximum of 28. Higher PI scores indicate a more positive perception of new technologies. In this study, the Personal Innovativeness scale was moderately consistent (Cronbach alpha = 0.64).

### 2.4. Procedure

The Monash University Human Research Ethics Committee (MUHREC) granted ethical approval for this study on August 17, 2022 (Project ID: 34959). A pilot study was conducted with a small group of participants who were familiar to the researchers. This allowed for the correction of survey display logic and the rewording of ambiguous questions. Participants were recruited through social media advertising on Facebook and Instagram, as well as mailing lists and online channels of relevant stakeholders such as the Monash University Accident Research Centre (MUARC) and the Department of Human-Centred Computing (HCC). Participation was entirely voluntary, with access to an explanatory statement outlining eligibility criteria and the chance to win one of five $100 (AUD) Myers Coles gift cards upon survey completion.

### 2.5. Data analysis

Data analysis was conducted using IBM SPSS Version 28. Histograms and boxplots were generated for continuous variables to identify and remove extreme outliers. This involved checking each variable for normal distribution and ensuring assumptions of heteroscedasticity and normality of residuals were met.

Out of 1,212 respondents, 155 were excluded due to incomplete surveys (less than 50% completed). Additionally, 11 duplicate entries were removed. This left 1,046 responses for analysis. However, due to their small sample size, nine non-binary participants were excluded from the gender analysis.

Table 1 presents descriptive statistics characterising the participants’ socio-demographic details (gender, age, education, income, ethnicity, and neurodiversity). Age responses were grouped into categories (<25 years, 26–30 years, 31–35 years, 36–40 years, 41–50 years, and 51+ years). The first two education groups (primary and intermediate) were combined due to limited responses in each category. Similarly, all income responses exceeding $125,000 were merged into a single category (> $125,001) due to sparsity in higher income brackets.

**Table 1:** Participant demographics (n = 1,046).

Category	Variable	% (*n*)
Gender	Men	52.5 (551)
	Women	47.5 (498)
	Non-binary	0.6 (7)
	Prefer not to say	0.2 (2)
Age	<25	15.7 (165)
	26–30	21.7 (228)
	31–35	19.3 (202)
	36–40	12 (126)
	41–50	13.5 (142)
	51+	17.7 (186)
Education	Primary / Intermediate	13.1 (138)
	VCE / HSC	16.1 (169)
	Technical / TAFE	17.7 (186)
	Diploma	18.4 (193)
	Undergraduate	20.9 (219)
	Postgraduate	13.3 (139)
	Other	0.5 (5)
Income	≤$25,000	4.5 (47)
	25,001-$50,000	9.6 (101)
	$50,001-$75,000	19.7 (207)
	$75,001-$100,000	21 (220)
	$100,001-$125,000	20.5 (215)
	>$125,001	20.3 (213)
	Prefer not to say	4.4 (46)
Ethnicity	Aboriginal or Torres Strait Islander	6.8 (71)
	Asian	4.9 (51)
	Black or African American	4.1 (43)
	Middle Eastern	3.0 (31)
	Native Hawaiian or Other Pacific Islander	4.6 (48)
	Caucasian	74.5 (781)
	Other	1.7 (18)
	Prefer not to say	0.6 (6)
Neurodiverse	Yes	16.1 (169)
	No	80.6 (845)
	Prefer not to say	3.3 (35)

Table 2 explores the relationship between current smartglass ownership and participant age, gender, and peer ownership using Chi-square tests.

**Table 2:** Relationship between smartglass ownership and gender, age group, and peer ownership (n = 1,037).

		**Ownership**		
		**Yes**	**No**	**Sig.**
		**% (*n*)**	**% (*n*)**	
**Gender**	Men	59.9 (326)	40.1 (218)	χ^2^ (1, *n* = 1037) = 0.684, p = 0.41
	Women	57.2 (282)	42.8 (211)	
	**Total**	58.6 (608)	41.4 (429)	
**Age**	<25	54.7 (88)	45.3 (73)	χ^2^ (5, *n* = 1,037) = 318.848, p <0.01 (Cramer’s V = 0.6)
	26–30	77 (174)	23 (52)	
	31–35	82.1 (165)	17.9 (36)	
	36–40	77.6 (97)	22.4 (28)	
	41–50	53.5 (76)	46.5 (66)	
	51+	4.4 (8)	95.6 (174)	
	**Total**	58.6 (608)	41.4 (429)	
**Peer Ownership**	**Knew someone**	95.6 (581)	17.2 (74)	χ^2^ (1, n = 1,037) = 659.568, p <0.01 (Phi = 0.8)
	**Did not know**	4.4 (27)	82.8 (355)	

Table 3 presents descriptive statistics regarding the types and frequency of use for various smartglass functions (photos, videos, music, and calls).

**Table 3:** Type of smartglass functionality and associated frequency of use (n = 607).

	Frequency % (*n*)				
Function	Never	Almost Never	Sometimes	Almost Every time	Frequently Use
**Photo**	2.7 (28)	4.1 (43)	17.4 (183)	18.6 (195)	15.1 (158)
**Video**	1.7 (18)	3.7 (39)	18.3 (192)	19.5 (205)	14.6 (153)
**Music**	1.9 (20)	3.1 (32)	19.6 (206)	18.1 (190)	15.2 (159)
**Calls**	1.6 (17)	4.2 (44)	19.9 (209)	16.9 (177)	15.3 (160)

Table 4 explores the relationship between self-reported daily smartglass use and engagement in dangerous/prohibited activities, analysed by participants’ gender and age group.

**Table 4:** Relationship between gender and age with daily use time, dangerous use, and prohibited use (n = 607).

		Avg. hours per day		Dangerous				Prohibited			
			Sig.	Yes	Maybe	No	Sig.	Yes	Maybe	No	Sig.
				% (*n*)	% (*n*)	% (*n*)		% (*n*)	% (*n*)	% (*n*)	
**Gender**	Men	4.00 (325)	χ^2^ (8, *n* = 607) = 14.054, p = 0.08	14.5 (47)	18.2 (59)	67.4 (219)	χ^2^ (2, *n* = 607) = 0.704, p = 0.70	15.4 (50)	11.4 (37)	73.2 (238)	χ^2^ (2, *n* = 607) = 1.537, p = 0.464
	Women	3.00 (282)		12.4 (35)	19.9 (56)	67.7 (191)		18.4 (52)	9.2 (26)	72.3 (204)	
**Age**	<25	4.00 (88)	χ^2^ (40, *n* = 607) = 75.524, p<0.001	33 (29)	45.5 (40)	21.6 (19)	χ^2^ (10, *n* = 607) = 124.496, p<0.001	36.4 (32)	34.1 (30)	29.5 (26)	χ^2^ (10, *n* = 607) = 137.493, p<0.001
	26–30	4.00 (173)		14.5 (25)	20.2 (35)	65.3 (113)		19.7 (34)	9.8 (17)	70.5 (122)	
	31–35	3.00 (165)		10.3 (17)	13.9 (23)	75.8 (125)		12.1 (20	4.2 (7)	83.6 (138)	
	36–40	2.00 (97)		5.2 (5)	7.2 (7)	87.6 (85)		3.1 (3)	6.2 (6)	90.7 (88)	
	41–50	2.00 (76)	Cramer’s V = 0.150	6.6 (5)	7.9 (6)	85.5 (65)	Cramer’s V = 0.320	11.8 (9)	1.3 (1)	86.8 (66)	Cramer’s V = 0.337
	51+	4.50 (8)		12.5 (1)	50 (4)	37.5 (3)		50 (4)	25 (2)	25 (2)	

Table 5 presents the results of correlation analyses and initial explorations of the association between the WEAR and PI scales. Descriptive statistics confirmed a significant violation of normality assumptions for both scales. Specifically, the WEAR scale scores were negatively skewed (clustered towards the right), while the PI scale scores were positively skewed (clustered towards the left). Therefore, a non-parametric test (Spearman’s R) was employed to assess the correlation between participants’ acceptance of new technologies (measured by the PI scale) and their perceptions and sentiments towards smartglasses (measured by the WEAR scale). The strength of this relationship was calculated by converting the squared R value to a percentage.

**Table 5:** WEAR scale and PI scale score mean, min & max, range, IQ, skewness & kurtosis.

	Md	Min, Max	Range	IQ	Skewness	Kurtosis	Sig.	Squared R-value
**WEAR**	51.00	14.00, 80.00	66.00	12.00	-.737	.663	*r* = .154, *n* = 1,006, p<0.001	39.24
**PI**	17.00	4.00, 28.00	24.00	6.00	.155	.085		

Table 6 explores the relationship between gender and ownership with WEAR scale scores using a Mann-Whitney U test. Additionally, a Kruskal-Wallis Test was conducted to explore the relationship between age groups and WEAR scale scores.

**Table 6:** Association between age group, gender and ownership with WEAR scale score (n = 1,009).

		WEAR score	
		Md (IQR)	Sig.
**Gender**	Men	51.00 (46.00–57.00)	U = 116622.50, z = -2.23, p < 0.001, r = 0.07
	Women	50.00 (42.00–56.00)	
**Age**	<25	52.00 (47.00–48.00)	χ2 (5, *n* = 1,009) = 110.729, p = < 0.001
	26–30	53.00 (49.00–59.00)	
	31–35	51.50 (47.00–56.00)	
	36–40	50.00 (46.00–55.00)	
	41–50	48.00 (37.25–55.00)	
	51+	39.50 (29.00–52.00)	
**Ownership**	Yes	53.00 (48.00–57.00)	U = 67712.00, z = -12.022, p < 0.001, r = 0.4
	No	44.00 (31.00–54.00)	

Table 7 further investigates this relationship for each WEAR scale item, focusing on differences between smartglass owners and non-owners.

**Table 7:** WEAR item results compared between smartglass owners and other participants (1 = strongly disagree, 6 = strongly agree).

WEAR scale		Ownership		Sig
		Yes	No	
Factor	Item	m (sd)	m (sd)	
Factor 1: Fulfilment of Aspirational Desires	I like what this device communicates about its wearer	3.91 (1.39)	3.27 (1.50)	U = 92664.00, z = -6.640, p < 0.001, r = 0.21
	I could imagine aspiring to be like the wearer of such a device	4.11 (1.33)	3.07 (1.61)	U = 77369.50, z = -10.067, p < 0.001, r = 0.32
	This device is consistent with my self-image	4.12 (1.30)	2.91 (1.54)	U = 68208.50, z = -12.125, p < 0.001, r = 0.40
	This device would enhance the wearer’s image	4.11 (1.31)	2.99 (1.52)	U = 73152.00, z = -11.020, p < 0.001, r = 0.35
	The wearer of this device would get a positive reaction from others	3.91 (1.32)	3.14 (1.40)	U = 86775, z = -7.975, p < 0.001, r = 0.25
	I like how this device shows membership to a certain social group	3.95 (1.44)	2.93 (1.51)	U = 76636.00, z = -10.213, p < 0.001, r = 0.32
	This device seems to be useful and easy to use	4.30 (1.31)	3.94 (1.30)	U = 103082.50, z = -4.336, p < 0.001, r = 0.14
	This device could help people	4.22 (1.36)	4.09 (1.34)	U = 116187.00, z = -1.363, p = 0.173
Factor 2: Absence of Social Fears	This device could allow its wearer to take advantage of people (R)	3.73 (1.47)	4.42 (1.32)	U = 90384.00, z = -7.158, p < 0.001, r = 0.23
	Use of this device raises privacy issues (R)	3.84 (1.40)	4.68 (1.37)	U = 80454.00, z = -9.408, p < 0.001, r = 0.30
	The wearer of this device could be considered rude (R)	3.53 (1.46)	4.07 (1.41)	U = 96651.50, z = -5.740, p < 0.001, r = 0.20
	Wearing this device could be considered inappropriate (R)	3.49 (1.48)	4.20 (1.40)	U = 89686.50, z = -7.301, p < 0.001, r = 0.23
	People would not be offended by the wearing of this device	4.01 (1.43)	2.95 (1.31)	U = 72723.00, z = -11.110, p < 0.001, r = 0.35
	This device would be distracting when driving (R)	3.75 (1.46)	4.21 (1.46)	U = 99443.500, z = -5.114, p < 0.001, r = 0.16

## 3. Results

### 3.1. Participant demographics

Table 1 presents the socio-demographic characteristics of the participants. Notably, 52.5% of participants (n = 551) were men. Undergraduate degrees represented the most common level of education attained, with a relatively even distribution across other levels (n = 219, 20.9%). Annual income was similarly distributed, with most participants earning between $50,000 and $125,000. Caucasian ethnicity was the most prevalent (n = 781; 74.5%), and 16.1% (n = 169) of participants self-identified as neurodiverse. To facilitate a more robust analysis, participants who did not select "men" or "women" were excluded. Following these adjustments, 1,037 participants were included in subsequent analyses.

### 3.2. Smartglass ownership

Over half of the participants (n = 608, 58.6%) reported owning smartglasses. Table 2 explores the relationship between smartglass ownership and gender, age group, and peer ownership using Chi-square tests.

While no significant association was found between gender and ownership, a strong relationship emerged between age group and ownership. Younger participants were significantly more likely to own smartglasses, with the 31–35 age group exhibiting the highest ownership rate (n = 165, 82.1%). A large effect size (Cramer’s V = 0.6) was present in this relationship [26].

Furthermore, a significant connection emerged between smartglass ownership and knowing other smartglass owners. Nearly all owners (n = 581; 95.6%) reported knowing other owners, demonstrating a strong positive relationship with a large effect size (Phi = 0.8) [26].

### 3.3. Frequency and types of smartglass use

Table 3 presents the frequency and types of smartglass use among owners (n = 607, excluding one participant with incomplete data). Participants reported using all device functions, including taking photos and videos, listening to music, and making/receiving calls. Notably, self-reported engagement across all functions was high, with participants indicating more frequent than infrequent use across all options. The open-ended question allowed participants to report additional types of use. Several participants frequently used audio and voice prompts, demonstrating the diverse applications of smartglasses beyond the preset functionalities.

Table 4 explores the relationships between participant gender, age group, and three key variables: 1) asking about average hours of smartglass use per day, measured by asking *How many hours per day do you typically use your smartglasses*? 2) asking about the frequency of dangerous use, measured by asking, *Have you ever used smartglasses in a manner that would be considered dangerous*, *such as while driving or cycling*? and 3) asking about prohibited use, measured by asking, *Have you ever used smartglasses in a prohibited manner*? *i*.*e*., *recording without consent or recording in a prohibited area*. A significant relationship was found between age groups and average daily use, with a small effect size. Younger participants reported spending more time using their smartglasses than older participants. No significant relationship was found between gender and dangerous or prohibited smartglass use. A significant relationship emerged between age groups and both types of anti-social use, with medium effect sizes. Younger participants were more likely to self-report engaging in both dangerous and prohibited behaviours with their smartglasses.

### 3.4. WEAR scale scores and their associations

There is a strong association between the WEAR scale scores and the PI scale scores, as shown in Table 5. Notably, PI scores explain nearly 40% of the variance in WEAR scores.

Non-parametric tests were conducted to investigate the associations between WEAR scale scores, age, gender, and ownership (Table 6). A significant, albeit very small, effect size (r = 0.07) was found for the relationship between gender and WEAR scale scores using a Mann-Whitney U test. A Kruskal-Wallis test revealed a significant difference in WEAR scale scores across the six age groups. Post-hoc tests identified the oldest age group (51+ years) as having significantly lower WEAR scores compared to all other groups. Additionally, a significant association emerged between participant ownership and WEAR scale scores. Smartglass owners exhibited higher scores than non-owners, indicating a medium to large effect size (r = 0.4) using Cohen’s [26] criteria.

Given the strong association between ownership and WEAR scale scores, further exploration was undertaken. Table 7 presents the mean scores and standard deviation for each WEAR scale item, comparing owners and non-owners.

Statistically significant differences were found for all WEAR scale items except for *"This device would help people*.*"* Within Factor 1: "Fulfilment of Aspirational Desires", smartglass owners displayed stronger agreement with all items, suggesting more positive sentiments regarding the device’s messaging. The largest mean differences were observed for *"This device is consistent with my self-image"* (r = 0.40), *"I could imagine aspiring to be like the wearer of such a device"* (r = 0.32), *"This device would enhance the wearer’s image"* (r = 0.35), and *"I like how this device shows membership to a certain social group"* (r = 0.32).

Regarding Factor 2: "Absence of Social Fears", participants who did not own smartglasses expressed greater agreement with concerns about the technology. The most significant mean differences were found for *"People would not be offended by the wearing of this device"* (r = 0.35) and *"Use of this device raises privacy issues"* (r = 0.30). These findings highlight an intriguing relationship between WEAR scale scores and participant ownership. Owners demonstrate more positive perceptions and lower anxiety towards smartglasses compared to non-owners. These insights offer valuable information for understanding the motivations and concerns associated with smartglass adoption, laying the groundwork for further research and development efforts in this field.

## 4. Discussion

This study investigated the use and social acceptability of commercially available smartglasses, such as Ray-Ban Stories by Meta. Building on prior research, our findings offer significant contributions to our understanding of user demographics, ownership rates, types of usage, and the relationship between technology acceptance and personal innovativeness and offer new considerations for managing expectations of appropriate smartglass use in public spaces.

Key insights:

Smartglass ownership

- There is a strong association between younger age groups and smartglass ownership, reflecting higher digital connectivity among younger Australians
- A significant majority (95.6%) of smartglass owners knew someone else who owned smartglasses, indicating the strong influence of social group norms on device ownership
- While gender did not significantly impact ownership, sociocultural expectations and the interplay of various power interests still influence wearable technology adoption

Smartglass use patterns and risks

- Younger users spend more time on average with their devices
- 13.5% of owners admit to dangerous use and 17% to anti-social use, underscoring the importance of including smartglasses in future regulatory frameworks, prioritising safety and intuitive design to mitigate risky behaviours
- Younger users are more likely to engage in dangerous or prohibited activities with smartglasses

Perceptions of smartglasses among non-users

- Owners expressed stronger agreement with items related to self-image and social status, viewing smartglasses as enhancing their self-perception and social connections.
- Non-owners expressed greater anxieties regarding privacy and fears of social disruption
- Younger participants and men were more likely to have higher WEAR scale scores, though gender differences were small.
- Owners were more likely to see smartglasses as a way to communicate their membership in specific social groups, reflecting a shift towards integrating technology and fashion.
- Both owners and non-owners recognized the potential benefits of smartglasses, but non-owners harboured concerns about the technology’s appropriateness and safety in public settings.
- Non-owners’ stronger concerns about anti-social outcomes and the potential for misuse highlight the need to address negative perceptions and societal impacts of smartglasses in public spaces.

### 4.1. Understanding smartglass ownership and usage

On face value, the findings may confirm the rising trend of smartglass adoption, with over half (58.6%) of participants reporting ownership. However, it is important to note that employing Facebook for participant recruitment likely explains the high number of smartglass owners due to the platform aligning content with the user’s interests.

We found a strong association between younger age groups and ownership, mirroring the observations of Zuidhof et al. [10]. Younger Australians exhibit demonstrably higher levels of digital connectivity than previous generations [27]. This increased exposure to technology through schooling, gaming, and mobile communications may contribute to their greater receptiveness towards emerging technologies like smartglasses. This aligns with Berkowsky et al. [28] and Olson et al. [29] who suggest that younger individuals are more likely to adopt and utilise a wider range of new technologies. However, it is important to acknowledge that the WEAR scale, specifically designed and tested on an 18–30 age group [23], may require further refinement to fully account for the broader age diversity present in this study.

Our findings also highlight the strong influence of social groups on device ownership. A significant majority (95.6%) of smartglass owners knew someone else who also owned smartglasses, compared to only 17.2% of non-owners who did so. This disparity underscores the significant impact of social group collective norms, which represent "prevailing codes of conduct that either prescribe or proscribe behaviours that members of a group can enact" [30].

Research examining the relationship between gender and wearable technologies reveals how sociocultural expectations are embedded in their availability and application. Wissinger [31] highlights the complex interplay of body, agency, commercial, and structural power interests and motives influencing the production, marketing, and consumer reception of these technologies. While this study’s finding that gender does not significantly impact smartglass ownership may suggest that utility, functionality, and convenience outweigh gender-related considerations, it does not necessarily imply the absence of gendered impacts. Further research with a more nuanced approach is needed to explore not only quantitative data but also qualitative insights regarding the perceptions and experiences of individuals of various genders within public spaces.

The findings suggest that shared understandings and perceptions of the device among younger age groups are associated with the likelihood of smartglass ownership. Research [15, 17] indicates that similar cognitive processes are involved in categorising people and objects, suggesting that a positive perception of a stereotypical user can promote the perception of a device as socially acceptable. Additionally, Antonetti and Maklan [32] proposed that the presence of stereotypical brand users influences an individual’s desire to own products associated with that brand. In the context of this study, high levels of shared group ownership among younger individuals may reflect positive perceptions of a stereotypical user they admire, leading to an increased likelihood of smartglass adoption. This underscores the need for targeted marketing and development strategies to cater to diverse user groups.

### 4.2. Smartglass usage patterns and potential risks: A closer look

The data reveals a variety of smartglass usage types, including photo and video capture, music listening, and communication, aligning with the diverse functionalities discussed by Iqbal & Campbell [8]. Younger users reported spending more time on average with their smartglasses compared to older users. Interestingly, all owners reported using photo, video, music, and call functions frequently or almost every time, highlighting the core functionalities that resonate with users. Although limited comparative research is available, a recent study [33] revealed that fewer than 10% of Ray-Ban Stories purchased since September 2021 are actively used on a monthly basis. Factors such as connectivity, battery life, and usability issues contribute to this low rate of active use.

Younger users were also more likely to report using their smartglasses in dangerous or prohibited ways, including driving or recording without consent. These findings align with research by Kaviani et al. [22] showing that younger users are more prone to engaging in risky and prohibited behaviours with smart mobile technologies. This underscores the importance of including smartglasses in future regulatory considerations and policies, particularly for high-risk environments. Manufacturers and developers should prioritise designing safe and intuitive interfaces and functionalities, incorporating age-specific safety features and warnings to mitigate risky behaviour among younger users.

Understanding smartglass usage patterns and potential risks is crucial for promoting responsible development and adoption. The findings highlight the need for age-specific considerations, user experience improvements, and regulatory frameworks that address emerging risks associated with this evolving technology. Smartglasses offer an opportunity to reframe how we understand children’s digital content engagement, moving beyond generic screen time limitations. Straker et al. [34] emphasise the need for a more nuanced approach, considering aspects like physical activity, sleep, and specific device usage patterns. Although the WEAR scale findings provide further insights into additional factors influencing social acceptability, along with a deeper understanding of potential barriers, further research, particularly qualitative studies, will be essential for developing effective interventions and ensuring a safe and beneficial smartglass experience for all users. Qualitative studies through focus groups can provide deeper insights into the motivations, attitudes, and behaviours surrounding smartglass use, allowing for targeted interventions and an improved understanding of the societal impact of smartglass adoption among young people.

### 4.3. Contrasting perceptions: Ownership, identity, and societal implications

The item-level analysis of the WEAR scale provided valuable insights into the specific aspects of smartglasses that resonate with users, as well as the key areas of concern that need to be addressed to promote safer use. This expands the call for research on user needs and concerns to consider the devices’ impacts on all members of the public. Owners expressed stronger agreement with items related to self-image, social status, and positive messaging associated with the device, reflecting the potential for self-enhancement and social connection discussed in the literature [8]. Conversely, non-owners expressed greater anxieties regarding privacy and potential social disruption, echoing the concerns raised by the ACCC inquiry and similar investigations [13, 35]. We found that participants who were initially more open to trying new technologies held more positive attitudes towards smartglasses. This factor should be considered when interpreting differences in WEAR item scores.

Age and gender were also significantly associated with WEAR scores. Younger participants and men were more likely to have higher scores, although the differences for gender were small.

Smartglass owners expressed significantly more positive feelings about how the device reflects their self-image and affiliation with specific social groups. They saw it as consistent with their personal identity, suggesting a positive contribution to self-perception. Similarly, owners were more likely to view smartglasses as a way to communicate their membership in a particular social group. This marks a shift from earlier research on the WEAR scale, which found that the appearance of wearables as assistive technology was key to their acceptability [19]. This shift likely reflects the increasing integration of technology and fashion. While owners held significantly more positive views than non-owners, there were no significant differences in perceptions of the device’s potential to help people. This suggests that despite recognising the potential benefits, non-owners still harbour concerns about the technology. This may be due to a lack of understanding about how users employ the device in public, as knowledge of its functions has been shown to reduce objections [36].

Non-owners expressed stronger concerns about privacy issues, potential anti-social outcomes, and the possibility of users taking advantage of others. They also agreed more strongly that wearing the device could be considered rude, inappropriate, or offensive. These findings highlight contrasting perceptions of appropriate and safe use between owners and non-owners. Previous research has documented how such frictions can manifest in negative reactions from bystanders [37], underscoring the need to address negative perceptions surrounding the device. This mirrors findings on drone technology in Australia [38] and necessitates a shift in research focus from consumer adoption to the social impacts of these devices in public spaces. The current consumer-centric approach often seeks to overcome adoption barriers rather than address broader social needs, leading to solutions that prioritise concealing cameras instead of informing people they are being filmed [39]. The risks and consequences associated with smartglasses pose significant challenges for urban governance, safety, and the overall experience of public spaces. A reorientation towards data-driven solutions that consider the concerns of all citizens and prioritise public safety is crucial. This will assist manufacturers and developers in designing technologies that are sensitive to the historical and cultural contexts in which they are used [40].

### 4.4. Navigating the future: Policy considerations for smartglasses

Smartglasses represent a rapidly evolving technology with the potential to revolutionise various aspects of our lives. Widespread adoption could have significant implications for social norms, privacy, and ethical considerations. Lessons from the past two decades of smartphone research demonstrate how personal portable technology fundamentally alters human interaction and public spaces. The regulatory lag in policy vis-à-vis enthusiastic smartphone adoption among Australians has contributed to negative habits such as dangerous, prohibited, and dependent use [22, 41]. This has resulted in increased risks to health, safety, and well-being, particularly for women and younger individuals.

Understanding public perception and utilisation of this technology is crucial for informing future development and ensuring ethical and responsible implementation, as emphasised by the Australian Privacy Act (1988) and similar regulations. Furthermore, the emergence of products like Microsoft Hololens, Magic Leap One, Vuzix Blade, and Nreal Light, featuring immersive Augmented Reality (AR) overlays, necessitates proactive policy development. Prior to widespread adoption, generating data on smartglass use is essential to inform policy and build the social and legislative framework upon which safe and ethical use can be defined.

This study’s insights provide valuable information for policymakers and stakeholders to address these challenges effectively. In Australia, the primary policy concern centres around the relationship between technology development, global providers like Alphabet (Google), Meta/Ray-Ban, and Snap Inc., and the inherent data harvesting practices of these companies. These concerns extend to emerging products in AR/VR. This concentration of ownership heightens concerns about privacy and surveillance in both public and private spaces. The ACCC and Attorney General’s Department, echoing critical reviews in Europe, raise privacy and security concerns related to smartglasses and other wearable products. These concerns include:

- Significant commercial partnerships underpinning product development.
- Unique privacy implications at the intersection of audiovisual, biometric, and location data.
- Potential for data-driven "hyper targeting" in advertising.

In light of these concerns, policymakers must closely monitor smartglass technology and establish frameworks that ensure privacy, security, and fundamental rights while promoting innovation. Further research is needed to examine types of use that present complex challenges, such as distinguishing between prescribed use for visually impaired wearers and non-prescribed use in prohibited or dangerous situations. Additionally, research should explore frameworks for mitigating risks and harms associated with unsafe or anti-social use, as well as the technology’s potential contributions to the efficiency and safety of pedestrians, safety for road users, such as using smartglasses as dash cams, and improved quality of experience within urban spaces, such as accessibility to services. The Drone Information Hub [42], established in Australia as a central government resource, offers a valuable model for smartglass policy development. This hub collates information at the intersection of privacy, infrastructure, and civil aviation policy domains, providing guidance for drone use. Notably, sectors driving drone uptake and policymaking, such as emergency services, health, and primary industry, are perceived as more acceptable by the Australian public compared to recreational use cases [38]. Generating similar awareness of sectoral use cases for smartglasses may be valuable for accelerating policy formation. By proactively addressing these challenges and learning from existing policy frameworks, policymakers can ensure the responsible development and adoption of this transformative technology.

## 5. Limitations

Our study’s findings require careful consideration due to several limitations. Firstly, relying on a convenience sample recruited online limits generalizability to the broader population. This approach potentially attracts individuals with internet access and positive sentiments towards internet-related technologies like Facebook, introducing bias. Additionally, focusing on Meta/Ray-Ban smartglasses and recruiting via Facebook Ads targeted users based on their interests further introduces bias by disproportionately attracting individuals already interested in smartglasses. The association between smartglass ownership and Facebook usage suggests these participants might be deeply ingrained in the Meta ecosystem, potentially influencing findings like higher WEAR scores among owners. Recognising these limitations encourages future investigations to utilise diverse recruitment methods and encompass broader demographics for a more comprehensive understanding of smartglasses. Future research could address this limitation by employing a multi-faceted recruitment strategy, including offline methods such as community outreach, partnerships with diverse organisations, and stratified sampling techniques to ensure a more representative sample of the general population.

Secondly, our study focused on smartglass ownership and usage within Australia. Our Facebook advertising was targeted to users residing in Australia, and survey participants had to meet the eligibility criteria of residing in Australia and providing their Australian state and postcode. Despite these procedures, we cannot guarantee the residential status of participants, however, the data may have limited generalizability to regions with different cultural contexts and technological landscapes than Australia. Furthermore, the cross-sectional design only offers a snapshot of attitudes and behaviours at one point in time, hindering the identification of causal relationships. We emphasise the need for further research that uses data collection methods beyond quantitative surveys. The ambiguity of the survey item assessing the frequency of function usage also needs acknowledgment. The response options for the question "How often do you use each function?" may have led to inconsistent interpretations among participants. As such, the data collected from this item might not accurately reflect participants’ actual usage patterns. Additionally, incorporating both a 6-point and a 7-point Likert scale might introduce response inconsistencies, potentially complicating the adjustment process for participants. To address these limitations, future research should implement a longitudinal study with a mixed-methods approach (i.e., that combines surveys and interviews and focus groups). This approach will provide a more comprehensive understanding of smartglass adoption and usage patterns across different contexts. The survey items should also be refined to ensure consistency in scale formats and clarifying potentially ambiguous items.

Third, the limitations of the WEAR Scale should also be acknowledged. Notably, measuring usefulness and ease of use within the single item, *“This device seems to be useful and easy to use”*. This approach is at odds with the well established Technology Acceptance Model. The TAM treated perceived usefulness and perceived ease of use as two distinct determining factors impacting attitudes, while attitudes determined the behavioural intention to use a technology [43]. While the TAM has proven to be one of the more popular models for explaining user adoption of new technologies, the framework was developed in 1986 [44], and the decision not to incorporate it was based on the availability of more contemporary models relevant to smartglass devices and the complex human and social factors influencing their social acceptability such as self-image, privacy, and social anxieties. Future research should also develop and validate a more comprehensive scale that captures the unique aspects of smartglass technology, such as privacy concerns and social acceptability. By doing so, it is anticipated that a more robust and nuanced tool for assessing smartglass adoption and usage will be created.

## 6. Conclusion

Despite these limitations, our study provides a comprehensive analysis of smartglass ownership, usage patterns, and perceptions, highlighting several critical insights for further research. Ownership is predominantly seen among younger age groups and influenced by peer norms, with younger users engaging more frequently and reporting higher instances of risky behaviours, emphasising the need for regulatory measures and age-specific safety features. The contrast between owners’ and non-owners’ perceptions underscores the importance of aesthetics, social status, and privacy concerns influencing acceptance of smartglass use in public space. Owners view smartglasses as enhancing self-image and social connections, while non-owners express significant anxieties about privacy and potential social disruptions. Overall, the study underscores the need for robust regulatory frameworks to ensure safe and beneficial use.

Future research should focus on qualitative insights to better understand the motivations and experiences of diverse groups negotiating smartglass use in public space, enabling the creation of technologies that are sensitive to the social and cultural contexts in which they are used.

## Supporting information

S1 File Consent form, WEAR scale items, personal innovativeness scale, perceptions and sentiments toward the use of smartglasses in public spaces survey. (DOCX)
