# Supplementary material for: Exploring social perceptions of everyday smartglass use in Australia
Source: PLoS One. 2024 Nov 1;19(11):e0313001. doi: 10.1371/journal.pone.0313001 (PMC11530075; doi:10.1371/journal.pone.0313001)
Supplement: S1 File — (DOCX) [file pone.0313001.s001.docx]

**Consent form.**

I have been asked to take part in the Monash University research project specified above.

I have read and understood the Explanatory Statement and I hereby consent to participate in this research project.

I understand that agreeing to take part means that I agree to complete an online survey related to my: demographic information (e.g., age, gender, ethnicity, etc.) and perceptions and sentiments towards smartglasses use.

I understand that any information I provide is confidential and that no information that could lead to the identification of any individual will be disclosed in any reports on the research project or to any other party. I also understand that my participation is voluntary, that I can choose not to participate in part or all of the online survey without being penalised or disadvantaged in any way. If I choose to withdraw, my data collected prior to this withdrawal will continue to be used and form part of the research project.

I have been informed that it is not possible to have access to individual feedback about my online survey data, but group results will be available from Monash University researchers upon request, once the research project has been completed.

I have also been informed that the information collected will be stored in a secure location at DHCC for a minimum of seven years, as required by Monash University regulations.

I consent to the above conditions and am willing to participate in the research project.

WEAR Scale items (Kelly & Gilbert, 2018)

Factor 1: Fulfillment of aspirational desires

1. I like what this device communicates about its wearer
2. I could imagine aspiring to be like the wearer of such a device
3. This device is consistent with my self-image
4. This device would enhance the wearer’s image
5. The wearer of this device would get a positive reaction from others
6. I like how this device shows membership to a certain social group
7. This device seems to be useful and easy to use
8. This device could help people

Factor 2: Absence of social fears

1. This device could allow its wearer to take advantage of people (R)
2. Use of this device raises privacy issues (R)
3. The wearer of this device could be considered rude (R)
4. Wearing this device could be considered inappropriate (R)
5. People would not be offended by the wearing of this device
6. This device would be distracting when driving (R)

Personal Innovativeness scale (Agarwal & Prasad, 1998)

1. If I heard about a new information technology, I would look for ways to experiment with it
2. Among my peers, I am usually the first to try out new information technologies
3. In general, I am hesitant to try out new information technologies (R)
4. I like to experiment with new information technologies

Perceptions and sentiments toward the use of smartglasses in public spaces survey

**End of Block: Explanatory statement & consent**

Please complete the following eligibility questions. If you are not eligible you will be sent to the end of the survey. We thank you for your time.

Are you aged 18 years or older?

- Yes (1)
- No (2)

*Skip To: End of Survey If Are you aged 18 years or older? = No*

Do you currently live in Australia?

- Yes (1)
- No (2)

*Skip To: End of Survey If Do you currently live in Australia? = No*

| Page Break |  |
| --- | --- |

You are eligible to participate in this survey. Please complete the following demographic questions.

| 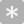 |
| --- |

What is your age (years)?

________________________________________________________________

What is your gender?

- Male (1)
- Female (2)
- Other (3) __________________________________________________
- Prefer not to say (4)

Location What is your residential state or territory?

- ACT (1)
- QLD (2)
- NSW (3)
- NT (4)
- SA (5)
- TAS (6)
- VIC (7)
- WA (8)

| 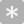 |
| --- |

Postcode Please enter your postcode

________________________________________________________________

Education What is your highest level of completed education?

- Primary school (1)
- Intermediate (Year 10 equivalent) (2)
- VCE / HSC (Year 12 equivalent) (3)
- Technical / TAFE (including trade certificate / apprenticeship) (4)
- Diploma (5)
- Undergraduate degree (6)
- Postgraduate degree (7)
- Other (8) __________________________________________________

Income What is your current yearly household income (AUD), before taxes?

- Less than or equal to $25,000 (1)
- $25,001 - $50,000 (2)
- $50,001 - $75,000 (3)
- $75,001 - $100,000 (4)
- $100,001 - $125,000 (5)
- $125,001 - $150,000 (6)
- $150,001 - $175,000 (7)
- $175,001 - $200,000 (8)
- $200,001 - $250,000 (9)
- More than $250,001 (10)
- Prefer not to say (11)

Ethnicity Which ethnicity grouping do you most closely align with?

- Aboriginal or Torres Strait Islander (1)
- Asian (2)
- Black or African American (3)
- Middle Eastern (4)
- Native Hawaiian or Other Pacific Islander (5)
- Caucasian (6)
- Other (Please specify) (7)
- Prefer not to say (8)

Neurodiversity Are you neurodiverse? (i.e., ADHD, Autism, Dyslexia)

- No (1)
- Yes (2)
- Prefer not to say (3)

**End of Block: Eligibility**

**Start of Block: Ray-ban Stories**

Q54 Before beginning please read this section carefully.

 The following image is of Ray-ban Stories smartglasses, a leading commercially available pair of smartglasses. 
 **It is important to note that commercially available smartglasses do not currently include augmented reality display (digital images displayed on the lens).**

 When connected to a smartphone, the eyewear’s in-built camera* allows wearers to take photos or video and upload directly to social media. The speakers and microphone enable calls and listening to music. All functions can be engaged hands-free or by using the eyewear's touch sensitive surface. Click here for more information.

*There is a colour indicator on the front of the glasses that lights up when camera recording is in use.

**End of Block: Ray-ban Stories**

**Start of Block: Current smartglasses use and familiarity**

| Page Break |  |
| --- | --- |

SG ownership The following questions are related to your familiarity with smartglasses

Smartglasses Prior to taking this survey, were you familiar with Ray-ban Stories or any other commercially available smartglasses? (*Note: this does not include Google Glass*)

- No (1)
- Somewhat familiar (2)
- Yes (3)

SG ownership Do you own a pair of commercially available smartglasses such as Ray-Ban Stories or Spectacles by Snap?

- Yes (1)
- No (2)

SG familiarity Do you personally know anyone that owns a pair of commercially available smartglasses such as Ray-ban Stories or Spectacles by Snap?

- Yes (1)
- No (2)

*Display This Question:*

*If Do you own a pair of commercially available smartglasses such as Ray-Ban Stories or Spectacles by... = Yes*

SG frequency On average, how many hours per day do you wear your smartglasses?

▼ Less than 1 (1) ... 9+ (9)

*Display This Question:*

*If Do you own a pair of commercially available smartglasses such as Ray-Ban Stories or Spectacles by... = Yes*

SG Use function What functionalities do you use when wearing your smartglasses?

|  | Never use (1) | Almost never (2) | Sometimes (3) | Almost everytime (4) | Frequently use (5) |
| --- | --- | --- | --- | --- | --- |
| Photo (1) |  |  |  |  |  |
| Video (2) |  |  |  |  |  |
| Music (3) |  |  |  |  |  |
| Calls (4) |  |  |  |  |  |
| Other (please provide details) (5) |  |  |  |  |  |

*Display This Question:*

*If Do you own a pair of commercially available smartglasses such as Ray-Ban Stories or Spectacles by... = Yes*

SG dangerous Have you ever used their functionality in a manner that would be considered dangerous? (i.e., while driving, cycling)

- No (1)
- Maybe (2)
- Yes (3)

*Display This Question:*

*If Do you own a pair of commercially available smartglasses such as Ray-Ban Stories or Spectacles by... = Yes*

SG prohibited Have you ever used them in a prohibited manner? (i.e., recording without consent, recording in a prohibited area)

- No (1)
- Maybe (2)
- Yes (3)

**End of Block: Current smartglasses use and familiarity**

**Start of Block: WEAR Scale**

| Page Break |  |
| --- | --- |

Q53 The following 14 questions are intended to gain an understanding of your perceptions and sentiments towards smartglasses.

W1 I like what this device communicates about its wearer

- Strongly disagree (1)
- Disagree (2)
- Somewhat disagree (3)
- Somewhat agree (4)
- Agree (5)
- Strongly agree (6)

W2 I could imagine aspiring to be like the wearer of such a device

- Strongly disagree (1)
- Disagree (2)
- Somewhat disagree (3)
- Somewhat agree (4)
- Agree (5)
- Strongly agree (6)

W3 This device is consistent with my self image

- Strongly disagree (1)
- Disagree (2)
- Somewhat disagree (3)
- Somewhat agree (4)
- Agree (5)
- Strongly agree (6)

W4 This device would enhance the wearer’s image

- Strongly disagree (1)
- Disagree (2)
- Somewhat disagree (3)
- Somewhat agree (4)
- Agree (5)
- Strongly agree (6)

W5 The wearer of this device would get a positive reaction from others

- Strongly disagree (1)
- Disagree (2)
- Somewhat disagree (3)
- Somewhat agree (4)
- Agree (5)
- Strongly agree (6)

W6 I like how this device shows membership to a certain social group

- Strongly disagree (1)
- Disagree (2)
- Somewhat disagree (3)
- Somewhat agree (4)
- Agree (5)
- Strongly agree (6)

W7 This device seems to be useful and easy to use

- Strongly disagree (1)
- Disagree (2)
- Somewhat disagree (3)
- Somewhat agree (4)
- Agree (5)
- Strongly agree (6)

W8 This device could help people

- Strongly disagree (1)
- Disagree (2)
- Somewhat disagree (3)
- Somewhat agree (4)
- Agree (5)
- Strongly agree (6)

W9 (R) This device could allow its wearer to take advantage of people

- Strongly disagree (1)
- Disagree (2)
- Somewhat disagree (3)
- Somewhat agree (4)
- Agree (5)
- Strongly agree (6)

W10 (R) Use of this device raises privacy issues

- Strongly disagree (1)
- Disagree (2)
- Somewhat disagree (3)
- Somewhat agree (4)
- Agree (5)
- Strongly agree (6)

W11 (R) The wearer of this device could be considered rude

- Strongly disagree (1)
- Disagree (2)
- Somewhat disagree (3)
- Somewhat agree (4)
- Agree (5)
- Strongly agree (6)

W12 (R) Wearing this device could be considered inappropriate

- Strongly disagree (1)
- Disagree (2)
- Somewhat disagree (3)
- Somewhat agree (4)
- Agree (5)
- Strongly agree (6)

W13 People would not be offended by the wearing of this device

- Strongly disagree (1)
- Disagree (2)
- Somewhat disagree (3)
- Somewhat agree (4)
- Agree (5)
- Strongly agree (6)

W14 (R) This device would be distracting when driving

- Strongly disagree (1)
- Disagree (2)
- Somewhat disagree (3)
- Somewhat agree (4)
- Agree (5)
- Strongly agree (6)

**End of Block: WEAR Scale**

**Start of Block: Personal Innovativeness**

| Page Break |  |
| --- | --- |

Q51 The following four questions are intended to gain an understanding of your current acceptance towards new technologies.

PI 1 If I heard about a new information technology, I would look for ways to experiment with it

- Strongly disagree (1)
- Disagree (2)
- Somewhat disagree (3)
- Neither agree nor disagree (4)
- Somewhat agree (5)
- Agree (6)
- Strongly agree (7)

PI 2 Among my peers, I am usually the first to try out new information technologies

- Strongly disagree (1)
- Disagree (2)
- Somewhat disagree (3)
- Neither agree nor disagree (4)
- Somewhat agree (5)
- Agree (6)
- Strongly agree (7)

PI 3 In general, I am hesitant to try out new information technologies

- Strongly disagree (1)
- Disagree (2)
- Somewhat disagree (3)
- Neither agree nor disagree (4)
- Somewhat agree (5)
- Agree (6)
- Strongly agree (7)

PI 4 I like to experiment with new information technologies

- Strongly disagree (1)
- Disagree (2)
- Somewhat disagree (3)
- Neither agree nor disagree (4)
- Somewhat agree (5)
- Agree (6)
- Strongly agree (7)

**End of Block: Personal Innovativeness**

**Start of Block: Final Block**

Q1 Thank you for completing this survey.

Would you like to enter the prize draw to win one of five $100 gift cards? (Please note, all duplicate entries will be deleted).

- Yes (please provide you email address) (1) __________________________________________________
- No (2)

Q2 Would you like to be contacted about future research at DHCC regarding this project?

- Yes (please provide your email address) (1) __________________________________________________
- No (2)

**End of Block: Final Block**
